# Sensitivity analysis of queueing models based on polynomial chaos approach

**DOI:** 10.1007/s40065-021-00344-y

**Published:** 2021-09-27

**Authors:** Lounes Ameur, Lahcene Bachioua

**Affiliations:** 1 Department of Technology, 20 August 1955 University of Skikda, Skikda, Algeria; 2 Department of Basic Sciences, Preparatory Year, University of Ha’il, P.O. Box 2440, Hail, Kingdom of Saudi Arabia

**Keywords:** 00A69, 00A71, 00A72, 05D40, 60K20, 60K25, 60J22, 65C05

## Abstract

Queueing systems are modeled by equations which depend on a large number of input parameters. In practice, significant uncertainty is associated with estimates of these parameters, and this uncertainty must be considered in the analysis of the model. The objective of this paper is to propose a sensitivity analysis approach for a queueing model, presenting parameters that follow a Gaussian distribution. The approach consists in decomposing the output of the model (stationary distribution of the model) into a polynomial chaos. The sensitivity indices, allowing to quantify the contribution of each parameter to the variance of the output, are obtained directly from the coefficients of decomposition. The proposed approach is then applied to M/G/1/N queueing model. The most influential parameters are highlighted. Finally several numerical and data examples are sketched out to illustrate the accuracy of the proposed method and compare them with Monte Carlo simulation. The results of this work will be useful to practitioners in various fields of theoretical and applied sciences.

## Introduction

Queueing models have been greatly studied and successfully applied in fabrication and production systems, service systems, telecommunication, transport, and the service industry. Typically, a queueing model is a simplified representation of the real-life system under consideration. In practice, input parameters of the model are not known exactly and are subject to uncertainty, because they are determined by insufficient statistical data. For these reasons, sensitivity analysis of queueing systems $({\mathbf {SAQS}})$ based on polynomial chaos $({\mathbf {PC}})$ has been developed. ${\mathbf {SAQS}}$ allows to analyze a queueing model by studying the impact of the variability of the model parameters on the output variable. Determining the parameters responsible for this variability makes it possible to take the necessary measures to reduce the variance of the output when the uncertainty on the output is important. Determining the least influential parameters can simplify the model by fixing the parameters to a nominal value of their interval of variation without significant repercussions on the output. Different approaches to sensitivity analysis have been proposed in the literature of Saltelli [18], Sobol [17] and Saltelli et al. [19, 20] and [21]. Approaches can be local or global. In this paper, we are interested in global methods, very frequent, which are based on the analysis of the variance of the output (ANOVA, ANalysis Of VAriance), Saltelli et al. [20] and Sobol [17], Jacques et al. [13]. The function of the model (here the stationary distribution $\pi$) is decomposed into a sum of functions of increasing dimensions Sobol [17]. This decomposition, called HDMR (High Dimensional Model Representation), allows to separate the effects of the different parameters, which are transmitted in the decomposition of the variance. To quantify the contribution of a parameter to the variance of the output, a sensitivity index is calculated. Sensitivity indices can sometimes be calculated formally, when the analytic form of the stationary distribution $\pi$ of the model is known and relatively simple. in our queueing model the stationary distribution $\pi$ is complex (nonlinear) and not analytically known. Unable to calculate these sensitivity indices, it is then necessary to estimate them Saltelli [18] and Gugole et al. [11]. Very often, the indices are estimated using Monte Carlo simulations. However, in some applications, this approach may require a high number of model evaluations, which makes the approach costly in computing time. Moreover, the measures are not always chosen freely because of the constraints of their implementation. In this case, the approach based on Monte Carlo simulations is no longer adapted. To overcome this problem, we will replace the queueing model by its analytical approximation based on the $({\mathbf {PC}})$ decomposition, which is less costly. The indices are then obtained directly from the expression of the coefficients of the polynomial decomposition. The $(\mathbf {CP})$ decomposition of the output has recently been used in an original way for the sensitivity analysis of Blatman and Sudret [6] and Blatman [5]. Wiener [23] has shown that any random variable following a normal distribution, and which possesses a finite variance, may be represented by a decomposition into Hermite polynomials, thus forming an orthogonal basis. Xiu and Karniadakis [25] have extended this decomposition to other types of distributions, which are associated with specific families of polynomials.

In this paper, we propose a numerical approach under uncertainty for M/G/1/N queueing model based on polynomials chaos, whose input parameters follow normal distributions; otherwise, in the case where the input parameters follow an arbitrary distribution, the transformation of these parameters to normal distributions is possible; see (Hamza et al. [12], Zhao and Ono [26]).

In this regard, we approach the expression of the stationary distribution $\pi$, the expected, and variance of different performance measures. To illustrate the accuracy of the proposed methods, we compare the obtained results with those obtained from repeated Monte Carlo simulations.

The rest of this paper is organized as follows: The embedded Markov chain of the M/G/1/N model is presented in Sect. 2. In Sect. 3, the expression of sensitivity indices is given. Their estimation based on decomposition in CP is also detailed. In Sect. 4, the proposed approach is applied to M/G/1/N queue, and numerical results of the computation of the stationary distribution and some performance measures under epistemic uncertainty are discussed in Sect. 5. Finally, Sect. 6 concludes the paper.

## Polynomial chaos expansion

Consider a computational model ${\mathcal {M}}$ whose input parameters are represented by a random vector $X=(X_{1},X_{2},\ldots ,X_{d})$, (the parameters $X_{i}, i=1, 2, \ldots , d$ are assumed to be independent real random variables) and the associated quantity of interest

1 $$\begin{aligned} Y = {\mathcal {M}}(X), \end{aligned}$$

the response *Y* is also uncertain, and its statistics are unknown and have to be estimated.

Denote by $(\Omega , {\mathcal {A}}, {\mathcal {P}})$ the probability space, where $\Omega$ is the set of all possible outcomes, ${\mathcal {A}}$ is a $\sigma$-algebra over $\Omega$, and ${\mathcal {P}}$ is a function ${\mathcal {A}}\rightarrow [0, 1]$ that gives a probability measure on ${\mathcal {A}}$ Consider an independent random vector $X=(X_{1},X_{2},\ldots , X_{d})$ that describes input uncertainties. The probability law of *X* may be defined by the probability density function

2 $$\begin{aligned} f_{X}(X)=\prod _{i=1}^{d}f_{i}(x_{i}), \end{aligned}$$

where $f_{i}(x_{i})$ is the marginal probability density of $X_{i}$.

Assuming that *Y* defined in (1) has a finite variance (which is a physically meaningful assumption when dealing with geotechnical systems), it belongs to the so-called Hilbert space of second-order random variables, which allows for the following representation [24]:

3 $$\begin{aligned} Y(X_{1}, \ldots , X_{d})= & {} a_{0}\psi _{0}+\sum _{i_{1}=1}^{\infty }a_{i_{1}}\psi _{1}(X_{i_{1}}) \nonumber \\&+ \sum _{i_{1}=1}^{\infty }\sum _{i_{2}=1}^{i_{1}}a_{i_{1}i_{2}}\psi _{2}(X_{i_{1}}, X_{i_{2}})\nonumber \\&+ \sum _{i_{1}=1}^{\infty }\sum _{i_{2}=1}^{i_{1}}\sum _{i_{3}=1}^{i_{2}} a_{i_{1}i_{2}i_{3}}\psi _{3}(X_{i_{1}}, X_{i_{2}}, X_{i_{3}})\nonumber \\&+ \sum _{i_{1}=1}^{\infty }\sum _{i_{2}=1}^{i_{1}}\sum _{i_{3}=1}^{i_{2}} \sum _{i_{4}=1}^{i_{3}} a_{i_{1}i_{2}i_{3}i_{4}}\psi _{3}(X_{i_{1}}, X_{i_{2}}, X_{i_{3}}, X_{i_{4}})\nonumber \\&+\cdots , \end{aligned}$$

where $\psi _{p}(.)$ is the polynomial chaos of order *p*.

After some rearranging (see [10]), this equation can be rewritten in a more convenient way as

4 $$\begin{aligned} Y=\sum _{j=0}^{\infty }{\widehat{b}}_{j}\psi _{j}(X), \end{aligned}$$

with deterministic coefficients ${\widehat{b}}_{j}$ which are unknown, to be estimated, $\psi _{j}$ are multivariate polynomials that depend of $X_{1},\ldots ,X_{d}$. It is given by

5 $$\begin{aligned} \psi _{\alpha }(X)=\psi _{\alpha _{1}, \ldots ,\alpha _{d}}(X)=\prod _{i=1}^{d}\phi _{\alpha _{i}}(X_{i}), \end{aligned}$$

where the multi-indices (also called tuples) $\alpha \in {\mathbf {N}}^{d}$ which are ordered lists of integers

6 $$\begin{aligned} \alpha =(\alpha _{1}, \ldots , \alpha _{d});\quad \alpha _{i} \in {\mathbf {N}} \end{aligned}$$

and $\phi _{\alpha _{i}}(X_{i})$ is a univariate polynomials depend on $X_{i}$.

The two-dimensional counterpart of Eq. (3) is rewritten, in a fully expanded form, as

7 $$\begin{aligned} Y(X)= & {} a_{0}\psi _{0}+a_{1}\psi _{1}(X_{1})+a_{2}\psi _{1}(X_{2})\nonumber \\&+ a_{11}\psi _{2}(X_{1},X_{1})+a_{12}\psi _{2}(X_{2}, X_{1})+a_{22}\psi _{2}(X_{2}, X_{2})\nonumber \\&+ a_{111}\psi _{3}(X_{1}, X_{1}, X_{1})+a_{211}\psi _{3}(X_{2}, X_{1}, X_{1})+a_{221}\psi _{3}(X_{2}, X_{2}, X_{1})+a_{222}\psi _{3}(X_{2}, X_{2}, X_{2})\nonumber \\&+ \cdots \end{aligned}$$

The Eq. (7) can be recast in terms of $\psi _{j}(.)$ as follows:

8 $$\begin{aligned} Y(X)= & {} {\widehat{b}}_{0}\psi _{0}+ {\widehat{b}}_{1}\psi _{1} +{\widehat{b}}_{2}\psi _{2}+{\widehat{b}}_{3}\psi _{3} +{\widehat{b}}_{4}\psi _{4}\nonumber \\&+ {\widehat{b}}_{5}\psi _{5}+{\widehat{b}}_{6}\psi _{6} +{\widehat{b}}_{7}\psi _{7}+{\widehat{b}}_{8}\psi _{8}+ {\widehat{b}}_{9}\psi _{9} +\cdots \end{aligned}$$

Now, for example the term $a_{211}\psi _{3}(X_{2}, X_{1}, X_{1})$ of Eq. (7) is identified with the term ${\widehat{b}}_{7}\psi _{7}$ of Eq. (8).

For a given degree *p*, the standard truncation scheme consists in selecting all polynomials, such that $|\alpha |=\sum _{i=1}^{d}\alpha _{i}\le p$ is smaller than *p*, i.e.,

9 $$\begin{aligned} {\mathcal {B}}^{d,p}=\{\alpha \in {\mathbf {N}}^{d}: |\alpha |\le p\}. \end{aligned}$$

The number of elements of a complete multivariate polynomial basis of degree *p* is

10 $$\begin{aligned} P_{d}^{p}=\text {card} ({\mathcal {B}}^{d,p})=\left( \begin{array}{c} p+d \\ d \\ \end{array} \right) =\frac{(p+d)!}{p!.d!}. \end{aligned}$$

The set of complete multivariate orthogonal basis $\{\psi _{k}\}_{k\ge 0}$ is consistent with the density of *X*. This family can be obtained by applying the Gram–Schmidt orthogonalization procedure to the canonical family of monomials $\{1,x^{2},x^{2},\ldots \}$. For standard distributions, the associated family of orthogonal polynomials is well known. If $X_{i}\sim U(-1, 1)$ has a uniform distribution over $[-1,1]$, the resulting family is that of so-called Legendre polynomials. If $X_{i}\sim {\mathcal {N}}(0,1)$ has a standard normal distribution with zero mean value and unit standard deviation, the resulting family is that of Hermite polynomials. The families associated with standard distributions are summarized in table (Xiu and Karniadakis [24]).

**Fig. 1:**
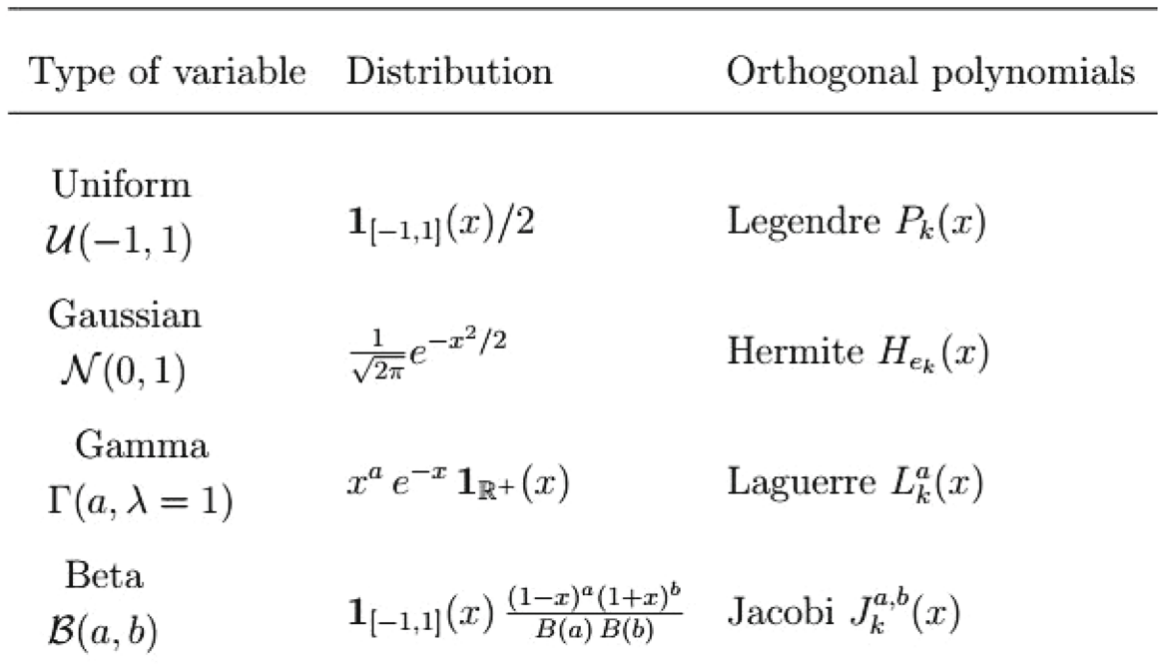
Classical families of orthogonal polynomials

### Remark

An input variable $X_{i}$ with a different distribution $F_{i}$ (continuous, strictly increasing) may be transformed to a random variable ${\widetilde{X}}_{i}$ (see [12]) with distribution *F* belonging to one of the classical families given in Fig. 1.

The series (4) is truncated by keeping terms up to a degree *p*

11 $$\begin{aligned} Y\approx \sum _{j=0}^{P_{d}^{p}-1}{\widehat{b}}_{j}\psi _{j}(X), \end{aligned}$$

where $P_{d}^{p}$ is given in (10). Note that $P_{d}^{p}$ increases polynomially with *d* and *p*. Thus, the number of terms in the Chao polynomial series, i.e., the number of coefficients to be computed, increases dramatically when $d > 10$, say. This complexity is referred to as curse of dimensionality, and there are advanced truncation schemes that allow one to bypass this problem (see [3, 14, 15]).

Now, let us give the Hermite polynomial chaos which is the very common example studied in the literature (the input random variables are ${\mathcal {N}}(0,1)$).

### The Hermite Chaos expansion

The original polynomial chaos, also termed as the homogeneous chaos, was first introduced by Wiener [22]. It employs the Hermite polynomials in terms of Gaussian random variables.

According to (3), the random response *Y* can be represented in the form

12 $$\begin{aligned} Y(X_{1}, \ldots , X_{d})= & {} a_{0}H_{0}+\sum _{i_{1}=1 }^{\infty }a_{i_{1}}H_{1}(X_{i_{1}}) \nonumber \\&+ \sum _{i_{1}=1}^{\infty }\sum _{i_{2}=1}^{i_{1}}a_{i_{1}i_{2}}H_{2}(X_{i_{1}}, X_{i_{2}})\nonumber \\&+ \sum _{i_{1}=1}^{\infty }\sum _{i_{2}=1}^{i_{1}}\sum _{i_{3}=1 }^{i_{2}}a_{i_{1}i_{2}i_{3}}H_{3}(X_{i_{1}}, X_{i_{2}}, X_{i_{3}}) \nonumber \\&+\cdots , \end{aligned}$$

where $H_{d}(X_{i_{1}},\ldots , X_{i_{d}})$ denote the Hermite polynomials of order *d* in terms of the multi-dimensional independent standard Gaussian random variables $X=(X_{i_{1}},\ldots , X_{i_{d}})$ with zero mean and unit variance. The above equation is the discrete version of the original Wiener polynomial chaos expansion, where the continuous integrals are replaced by summations. The general expression of the Hermite polynomials is given by

13 $$\begin{aligned} H_{d}(X_{i_{1}},\ldots , X_{i_{d}})=\exp \left( \frac{1}{2} X^{{\mathrm{T}}} X\right) (-1)^{n}\frac{\partial ^{n}}{\partial X_{i_{1}} \ldots \partial X_{i_{d}}}\exp \left( \frac{-1}{2} X^{{\mathrm{T}}}\xi \right) . \end{aligned}$$

For example, the one-dimensional Hermite polynomials are

14 $$\begin{aligned} \psi _{0}=1; \psi _{1}=X; \psi _{2}=X^{2}-1; \psi _{3}=X^{3}-3X; \ldots \end{aligned}$$

For notational convenience, Eq. (12) can be rewritten as

15 $$\begin{aligned} Y(X)=\sum _{j=0}^{\infty }{\widehat{b}}_{j}\psi _{j}(X), \end{aligned}$$

where there is a one-to-one correspondence between the functions $H_{d}(X_{i_{1}},\ldots , X_{i_{d}})$ and $\psi _{j}(X)$, and also between the coefficients ${\widehat{b}}_{j}$ and $a_{i_{1}\ldots i_{r}}$. In Eq. (12), the summation is carried out according to the order of the Hermite polynomials, while in Eq. (15), it is simply a re-numbering with the polynomials of lower order counted first. For clarity, the two-dimensional expansion is shown here, both in the fully expanded form [see Eq. (12)]

16 $$\begin{aligned} Y(X)=a_{0}H_{0}+a_{1}H_{1}(X_{1})+a_{2}H_{1}(X_{2}) +a_{11}H_{2}(X_{1},X_{1})+a_{12}H_{2}(X_{2},X_{1}) +a_{22}H_{2}(X_{2},X_{2})+\cdots ; \nonumber \\ \end{aligned}$$

and the simplified form [see Eq. (15)]

17 $$\begin{aligned} Y(X)= & {} {\widehat{b}}_{0}\psi _{0}+{\widehat{b}}_{1}\psi _{1} +{\widehat{b}}_{2}\psi _{2}+{\widehat{b}}_{3}\psi _{3} +{\widehat{b}}_{4}\psi _{4}+{\widehat{b}}_{5}\psi _{5}+\cdots \nonumber \\= & {} {\widehat{b}}_{0}+{\widehat{b}}_{1}X_{1}+{\widehat{b}}_{2}X_{2} +{\widehat{b}}_{3}(X_{1}^{2}-1)+{\widehat{b}}_{4}(X_{1}X_{2}) +{\widehat{b}}_{5}(X_{2}^{2}-1)+\cdots \end{aligned}$$

#### Example 2.1

The two-dimensional hermite polynomial chaos for different degree *p* is given by (Table 1).

**Table 1:** Two-dimensional Hermite-polynomial chaos, ${d}=2$

*j*	Order of the polynomial chaos	*j*th polynomial chaos $\psi _{j}$
0	${p}=0$	1
1	${p}=1$	$X_{1}$
2		$X_{2}$
3	${p}=2$	$X_{1}^{2}-1$
4		$X_{1}X_{2}$
5		$X_{2}^{2}-1$
6	${p}=3$	$X_{1}^{3}-3X_{1}$
7		$X_{1}^{2}X_{2}-X_{2}$
8		$X_{1}X_{2}^{2}-X_{1}$
9		$X_{2}^{3}-3X_{2}$
10	${p}=4$	$X_{1}^{4}-6X_{1}^{2}+3$
11		$X_{1}^{3}X_{2}-3X_{1}X_{2}$
12		$X_{1}^{2}X_{2}^{2}+X_{1}^{2}-X_{2}^{2}+1$
13		$X_{1}X_{2}^{3}-3X_{1}X_{2}$
14		$X_{2}^{4}-6X_{2}^{2}+3$

#### Remark 2.2

Ghanem and Spanos [10] give the three-dimensional and four-dimensional hermite polynomial chaos for different degree *p*.

### Calculation of polynomial chaos coefficients

Several approaches have been developed to estimate the coefficients of the CP that can be classified as intrusive (Xiu and Karniadakis [25]) or non-intrusive (Berveiller et al. [4]). Among the non-intrusive approaches, we quote: the method of projection, the method of regression, and the method of the least squares which will be retained in the sequel, for its simplicity of implementation.

We denote by *B* the vector of the coefficients ${\widehat{b}}_{j}, j=0,\ldots , d$, to be determined. Let *U* be the vector containing the *N* samples of the output *Y* and *Z* the matrix containing the polynomials $\psi _{j}$

$$\begin{aligned} Z=\left( \begin{array}{ccc} \psi _{0}(X_{1}^{(1)},\ldots ,X_{n}^{(1)}) &{} \dots &{} \psi _{d}(X_{1}^{(1)},\ldots ,X_{n}^{(1)}) \\ \vdots &{} \vdots &{} \vdots \\ \psi _{0}(X_{1}^{(N)},\ldots ,X_{n}^{(N)}) &{} \dots &{} \psi _{d}(X_{1}^{(N)},\ldots ,X_{n}^{(N)}) \\ \end{array} \right) \end{aligned}$$

with $X_{k}^{(i)}$ represents the *i*th sample of $X_{k}$. The output vector *U* is given by $U=ZB$. The estimator of the vector *B*, denoted ${\widehat{B}}$, is given by the least-squares method

18 $$\begin{aligned} {\widehat{B}}=(Z^{{\mathrm{T}}}Z)^{-1}Z^{{\mathrm{T}}}U. \end{aligned}$$

The estimated output ${\widehat{U}}=({\widehat{Y}}^{(1)},\ldots ,{\widehat{Y}}^{(n)})^{{\mathrm{T}}}$ is thus given by the following expression:

19 $$\begin{aligned} {\widehat{U}}=Z{\widehat{B}}. \end{aligned}$$

## Sensitivity analysis based on polynomial chaos

Consider a mathematical model, consisting of a set of random input variables, a deterministic function, and a set of random output variables(or responses). We write this model in the following form:

20 $$\begin{aligned} Y: {\mathbb {R}}^{d}\longrightarrow {\mathbb {R}}^{N}, X \longrightarrow Y=Y(X)=(Y_{1}(X),Y_{2}(X),\ldots ,Y_{N}(X)), \end{aligned}$$

where

21 $$\begin{aligned} Y_{\ell }: {\mathbb {R}}^{d}\longrightarrow {\mathbb {R}}, X \longrightarrow Y_{\ell }=Y_{\ell }(X),\quad \ell =1,\ldots ,N. \end{aligned}$$

The function *Y* is the response of the model, and the set of input variables $X=(X_{1},X_{2},\ldots ,X_{d})$ groups all the entities considered random in the model. Quantities $X_{i},i=1,\ldots ,p$ are assumed to be independent random variables.

The purpose is to study the sensitivity analysis of each component $Y_{\ell }$. An indicator of the influence of the parameter $X_{i}$ on the output $Y_{\ell }$ denoted $S_{i}^{\ell }$ (see Sobol [17]) is defined by

22 $$\begin{aligned} S_{i}^{\ell }=\frac{{\mathbb {V}}({\mathbb {E}}(Y_{\ell }/ X_{i}))}{{\mathbb {V}}(Y_{\ell })},\quad \ell =1,2,\ldots ,N. \end{aligned}$$

This index is called first-order sensitivity index of Sobol [17]. It quantifies the sensitivity of the output $Y_{\ell }$ to the input variable $X_{i}$, or the part of the variance of $Y_{\ell }$ due to the variable $X_{i}$.

The sensitivity index $S_{i}^{\ell }$ is between 0 and 1; more the index is elevated (close to 1), more the variable $X_{i}$ is influential (will have importance).

The variance of the model with independent inputs can be decomposed as follows:

23 $$\begin{aligned} V^{\ell }=\sum _{i=1}^{n}V_{i}^{\ell }+\sum _{1\le i< j \le n}V_{ij}^{\ell }+\cdots +V_{1\ldots n}^{\ell } \end{aligned}$$

with

24 $$\begin{aligned}&V_{ij}^{\ell }= V^{\ell }({\mathbf {E}}(Y^{\ell }/X_{i}, X_{j}))-V_{i}^{\ell }-V_{j}^{\ell } \nonumber \\&\quad \dots \nonumber \\&V_{1\ldots n} = V^{\ell }(Y)- \sum _{i=1}^{n}V_{i}^{\ell }-\sum _{1\le i< j\le n}V_{ij}^{\ell }-\cdots -\sum _{1\le i_{1}<\cdots \le i_{n-1}\le n}V_{i_{1}\ldots i_{n-1}}^{\ell }; \end{aligned}$$

based on this decomposition (see Sobol [17]), the sensitivity indices of second order are given by

25 $$\begin{aligned} S_{ij}^{\ell }=\frac{V_{ij}^{\ell }}{V^{\ell }},\quad \ell =1,\ldots ,N. \end{aligned}$$

These indices express the sensitivity of the variance of $Y_{\ell }$ to the interaction of the variables $X_{i}$ and $X_{j}$, that is, the sensitivity of $Y_{\ell }$ to the variables $X_{i}$ and $X_{j}$ which is not taken into account in the effect of the variables alone. The higher order sensitivity indices are defined on the same principle. The total sensitivity index (see Gugole et al. [11]), Cacuci et al. [7] which expresses the total sensitivity of the variance *Y* to a variable $X_{i}$, that is, sensitivity to this variable in all its forms (sensitivity to the variable alone and sensitivity to the interactions of this variable with other variables), is defined as the sum of all the sensitivity indices relative to the variable $X_{i}$

26 $$\begin{aligned} S_{T_{i}}^{\ell }=S_{i}^{\ell }+\sum _{j\ne i}S_{ij}^{\ell }+ \sum _{j\ne i, k\ne i, j< k}S_{ijk}^{\ell }+\cdots \end{aligned}$$

For example, for a model with three input variables, we have

$$\begin{aligned} S_{T_{1_{}}}=S_{1}^{\ell }+S_{12}^{\ell }+S_{13}^{\ell }+S_{123}^{\ell }, \quad \ell =1,\ldots ,N. \end{aligned}$$

Since the model function is not known analytically, it is necessary to estimate these indices. In the literature, different estimation methods have been proposed by Cukier et al. (1978). The approach frequently used for its simplicity of implementation is based on simulations of Monte Carlo. However, the number of evaluations of the model can become high and costly in calculation time. To avoid this disadvantage, the model is replaced by its decomposition on $({\mathbf {PC}})$, which is an analytic representation in an orthogonal polynomial basis. The sensitivity indices are obtained directly from the PC decomposition coefficients.

### Estimation of sensitivity indices

According to (11), each component $Y_{\ell }$ of *Y* can be developed as follows:

27 $$\begin{aligned} Y_{\ell }\approx \sum _{j=0}^{P_{d}^{p}-1}{\widehat{b}}_{j}^{\ell } \psi _{j}(X_{1},\ldots ,X_{d}),\quad \ell =1,\ldots ,N. \end{aligned}$$

After determining the coefficients of the CP and by reordering the terms of (27) according to the parameters on which they depend, we obtain the following decomposition:

28 $$\begin{aligned} Y_{\ell }= & {} {\widehat{b}}_{0}^{\ell }+\sum _{k=1}^{d}\sum _{j\in \varGamma _{k}}{\widehat{b}}_{j}^{\ell }\psi _{j}(X_{k})+\sum _{1\le k_{1}\le k_{2}\le d }\sum _{j\in \varGamma _{k_{1},k_{2}}}{\widehat{b}}_{j}^{\ell }\psi _{j}(X_{k_{1}},X_{k_{2}})+\cdots \nonumber \\&+ \sum _{1\le k_{1} \le \cdots \le k_{s}\le d }\sum _{j\in \varGamma _{k_{1},\ldots ,k_{s}}}{\widehat{b}}_{j}^{\ell }\psi _{j}(X_{k_{1}},\ldots ,X_{k_{s}})+\cdots +\sum _{j\in \varGamma _{1,\ldots ,P_{d}^{p}}}{\widehat{b}}_{j}^{\ell }\psi _{j}(X_{k_{1}},\ldots ,X_{k_{d}}) \end{aligned}$$

with $\varGamma _{k_{1},\ldots ,k_{s}}$ the set of multi-indices j which corresponds to the polynomials dependent only on variables $X_{k_{1},\ldots ,k_{s}}$.

According to expression (28), the estimate of the first-order sensitivity index $\widehat{S_{i}^{\ell }}$ is given by

29 $$\begin{aligned} \widehat{S_{i}^{\ell }}=\frac{\sum _{j\in \varGamma _{i}}\widehat{b_{j}^{\ell }}^2{\mathbf {E}} (\psi _{j}^2(X_{i}))}{\sum _{j=1}^{P_{d}^{p}} \widehat{b_{j}^{\ell }}^2{\mathbf {E}}(\psi _{j}^2(X_{i},\ldots ,X_{d}))},\quad \ell =1,\ldots ,N, \end{aligned}$$

where $b_{j}^{\ell }$ are the coefficients estimated by (18) and the set $\varGamma _{i}$ corresponds to the polynomials $\psi _{j}$ depending only on $X_{i}$.

In the same way, the estimate of the sensitivity index $\widehat{S_{ir}^{\ell }}$ due to the interaction between the variables $X_{i}$ and $X_{r}$ is given by

30 $$\begin{aligned} \widehat{S_{ir}^{\ell }}=\frac{\sum _{j\in \varGamma _{ir}}\widehat{b_{j}^{\ell }}^2{\mathbf {E}}(\psi _{j}^2 (X_{i},X_{r}))}{\sum _{j=1}^{P_{d}^{p}} \widehat{b_{j}^{\ell }}^2{\mathbf {E}}(\psi _{j}^2(X_{i},\ldots ,X_{d}))},\quad \ell =1,\ldots ,N. \end{aligned}$$

Moreover, the estimated total sensitivity index is written in this form (see [11])

31 $$\begin{aligned} \widehat{S_{T_{i}}^{\ell }}=\frac{\sum _{j\in \varGamma _{i^{+}}}\widehat{b_{j}^{\ell }}^2{\mathbf {E}} (\psi _{j}^2(X_{i}))}{\sum _{j=1}^{P_{d}^{p}} \widehat{b_{j}^{\ell }}^2{\mathbf {E}}(\psi _{j}^2(X_{i},\ldots ,X_{d}))},\quad \ell =1,\ldots ,N. \end{aligned}$$

The CP forms an orthogonal basis, which guarantees the uniqueness of the decomposition and hence the uniqueness of the sensitivity indices (29)–(31).

## Application to the queueing model

In this section, we consider the PC expansion for propagating the uncertainty in performance measures of the M/GI/1 queue, and study the influence of each input parameter and their possible interactions onto the output measures.

### The M/G/1 queue

We consider an M/G/1 queue with finite capacity ${\mathbf {N}}$, where clients are served according to the discipline ${\mathbf {FIFO}}$. The customers arrive according to a Poisson process with rate $\lambda$. The service times is a sequence of random variables distributed according to a general common distribution G(t) of mean $1/\mu$. In the sequel, we discuss an example motivating the practical features of this queueing model [2].

Storage Area Networks (SAN) have become one of the most widely used solutions for high-performance enterprise storage applications. However, the infrastructures of SANs are very complex and varied among different vendors. It is important to understand the behavior of these SANs in order of enhancing performance of the applications that run on them. The main idea of this paper is to understand the behavior of a server-SAN system that manages multiple applications stored in the same volume. The aim is modeling a typical workload of a small company which consists of one license of database system software and shares that same license for multiple databases. These multiple databases may be used potentially for diverse applications. The objective is then to see how the system would behave when managing queries from different types of databases at the same time. An M/G/1 Queueing Model has been used by [Emmanuel Arzuaga and David R. Kaeli] to analyze the system and they give a report set of experiments which are composed of two different database workloads based on the TPC-C and TPC-H benchmarks; they also show that the model can be used to anticipate the impact of growth in the arrival rate on the behavior of the system. This model has the potential of predicting the system behavior when adding more workloads to the mix.

Let $Q_{n}=Q(t_{n}^{+})$ be the number of customers in the system just after the departure of the *n*th customers; it is clear that $Q_{n}$ is an induced Markov chain with space state ${0, 1,\ldots , N - 1}$. The one-step transition matrix associated of the Markov chain $Q_{n}$ (see [9]) is given by

32 $$\begin{aligned} P=\left( \begin{array}{ccccccc} a_{0} &{} a_{1} &{} a_{2} &{} a_{3} &{} \cdots &{} a_{N-2} &{} 1-\sum _{k=0}^{N-2}a_{k} \\ a_{0} &{} a_{1} &{} a_{2} &{} a_{3} &{} \cdots &{} a_{N-2} &{} 1-\sum _{k=0}^{N-2}a_{k} \\ 0 &{} a_{0} &{} a_{1} &{} a_{2} &{} \cdots &{} a_{N-3} &{} 1-\sum _{k=0}^{N-3}a_{k} \\ 0 &{} 0 &{} a_{0} &{} a_{1} &{} \cdots &{} a_{N-4} &{} 1-\sum _{k=0}^{N-4}a_{k} \\ 0 &{} 0 &{} 0 &{} a_{0} &{} \cdots &{} a_{N-5} &{} 1-\sum _{k=0}^{N-5}a_{k} \\ \vdots &{} \vdots &{} \ddots &{} \vdots &{} \vdots &{} \vdots &{} \vdots \\ 0 &{} 0 &{} 0 &{} 0 &{} \cdots &{} a_{0} &{} 1-a_{0} \\ \end{array} \right) , \end{aligned}$$

where

33 $$\begin{aligned} a_{k}=\int _{0}^{\infty }\frac{(\lambda t)^{k}}{k!} \exp (-\lambda t) \text {d} G(t),\quad k= 0,\ldots , N-2. \end{aligned}$$

A service-time distribution (*G*),  having a finite *i*th moment $m^{(i)}$, such that its coefficient of variation CV, can be approximated by a phase type distribution PH. Therefore, we use M/PH/1/N queue as approximation to M/GI/1/N queue

1. if the coefficient of variation of the service-time distribution $\text {CV}=0$, the distribution is deterministic;
2. if the coefficient of variation of the service-time distribution $\text {CV}=1$, the PH distribution is exponential;
3. if the coefficient of variation of the service-time distribution $\text {CV}<1$, the PH distribution is hypoexponential;
4. if the coefficient of variation of the service-time distribution $\text {CV}>1$, the PH distribution is hyperexponential.

To test the efficiency of the polynomial chaos decomposition and explore particularly the sensitivity of the input parameters on the considered performance measures. We limit our study with particular case when the service time is exponential and we provide a variety of numerical results for various performance measures and the results are exhibited via tables and graphs. All the calculations are done on Matlab Software Package. In the next section, we will discuss a functional approach based on PC expansion for computing the epistemic uncertainty in stationary distribution $\pi (\lambda ,\mu )=(\pi _{1}(\lambda ,\mu ),\ldots ,\pi _{N}(\lambda ,\mu ))$, due to epistemic uncertainties in $\lambda$ and $\mu$.

In our numerical computation, we set $X_{1}= \lambda$ the arrival rate, and $X_{2}=\mu$ the service rate, which are supposed random variables of normal distribution, with mean ${\overline{\lambda }}$, ${\overline{\mu }}$, respectively. The output of the model represents the stationary distribution $\pi (\lambda ,\mu )$. In the M/M/1 system, we consider the following situations: ${\overline{\lambda }}={\overline{\mu }}=2/3$, $\sigma _{1}=\sigma _{2}=0.1$ and $N=4$, The aim is to study the influence of the $\lambda$ and $\mu$ parameters on the variance of the response of the model $\pi$, and we apply the approach proposed in the Sect. 2.

Each component of the output of the model $\pi$ is decomposed into PC (27)

34 $$\begin{aligned} \pi _{\ell }\approx \sum _{j=0}^{9}{\widehat{b}}_{j}^{\ell }\psi _{j}(\lambda ,\mu ),\quad \ell =1,\ldots ,4. \end{aligned}$$

The number of parameters $d=2$, and the desired degree for the polynomials is fixed to $p=3$. According to (10), the number of coefficients ${\widehat{b}}_{j}^{\ell }, \ell =1,\ldots ,4$ is equal to 10. $\{\psi _{j}\}_{0\le j \le 9}$ form an orthonormal Hermite polynomial basis: $\psi _{0}(\lambda ,\mu )=1$, $\psi _{1}(\lambda ,\mu )=\lambda$, $\psi _{2}(\lambda ,\mu )=\mu$, $\psi _{3}(\lambda ,\mu )=\lambda ^2 - 1$, $\psi _{4}(\lambda ,\mu )=\lambda \mu$, $\psi _{5}(\lambda ,\mu )=\mu ^2 - 1$, $\psi _{6}(\lambda ,\mu )=\lambda ^3 - 3\lambda$, $\psi _{7}(\lambda ,\mu )=\mu (\lambda ^2- 1)$, $\psi _{8}(\lambda ,\mu )=\lambda (\mu ^2 -1)$, $\psi _{9}(\lambda ,\mu )=\mu ^3 -3\mu$.

**Fig. 2:**
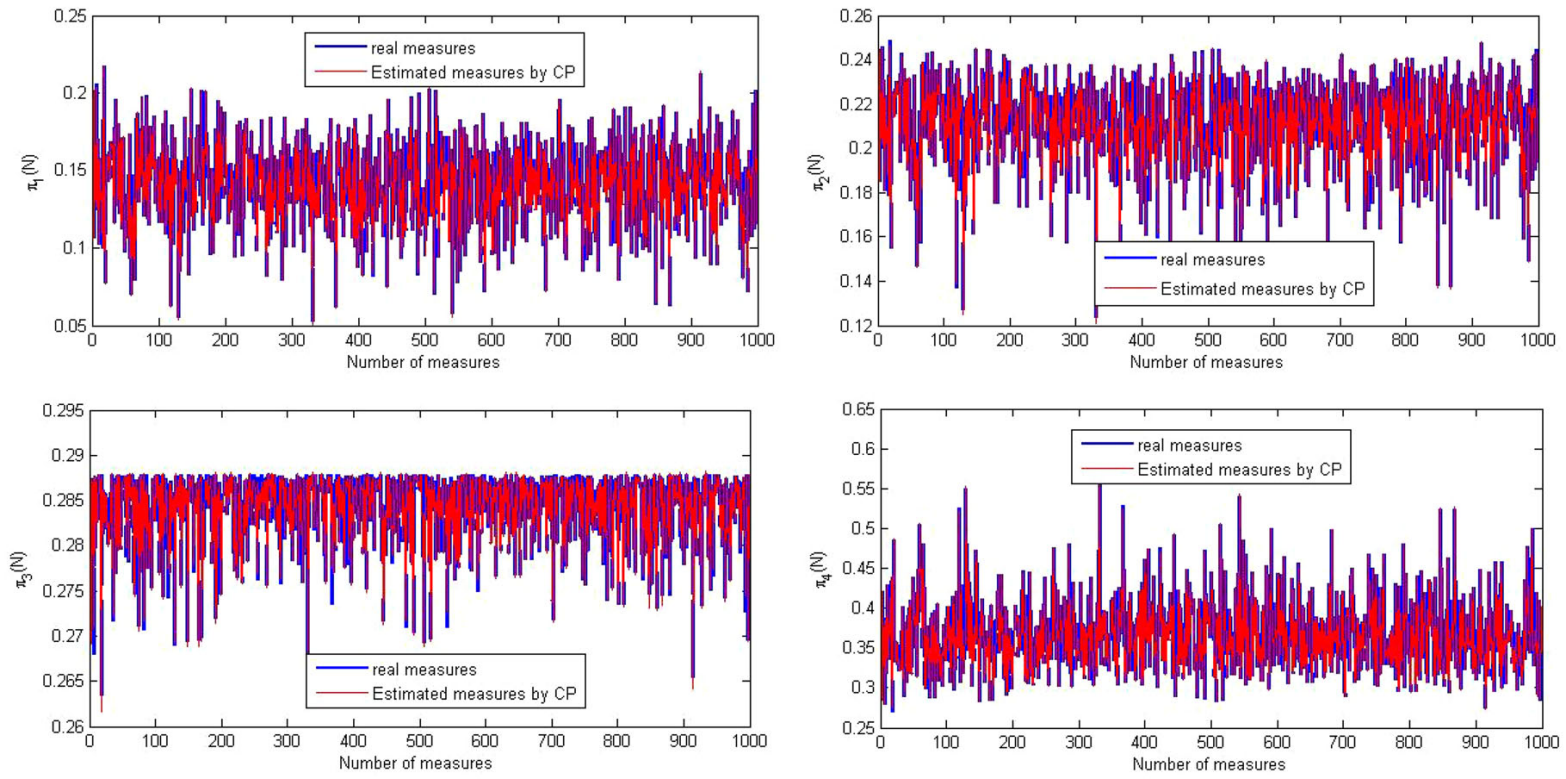
Evolutions of the real output and estimated output by PC

**Fig. 3:**
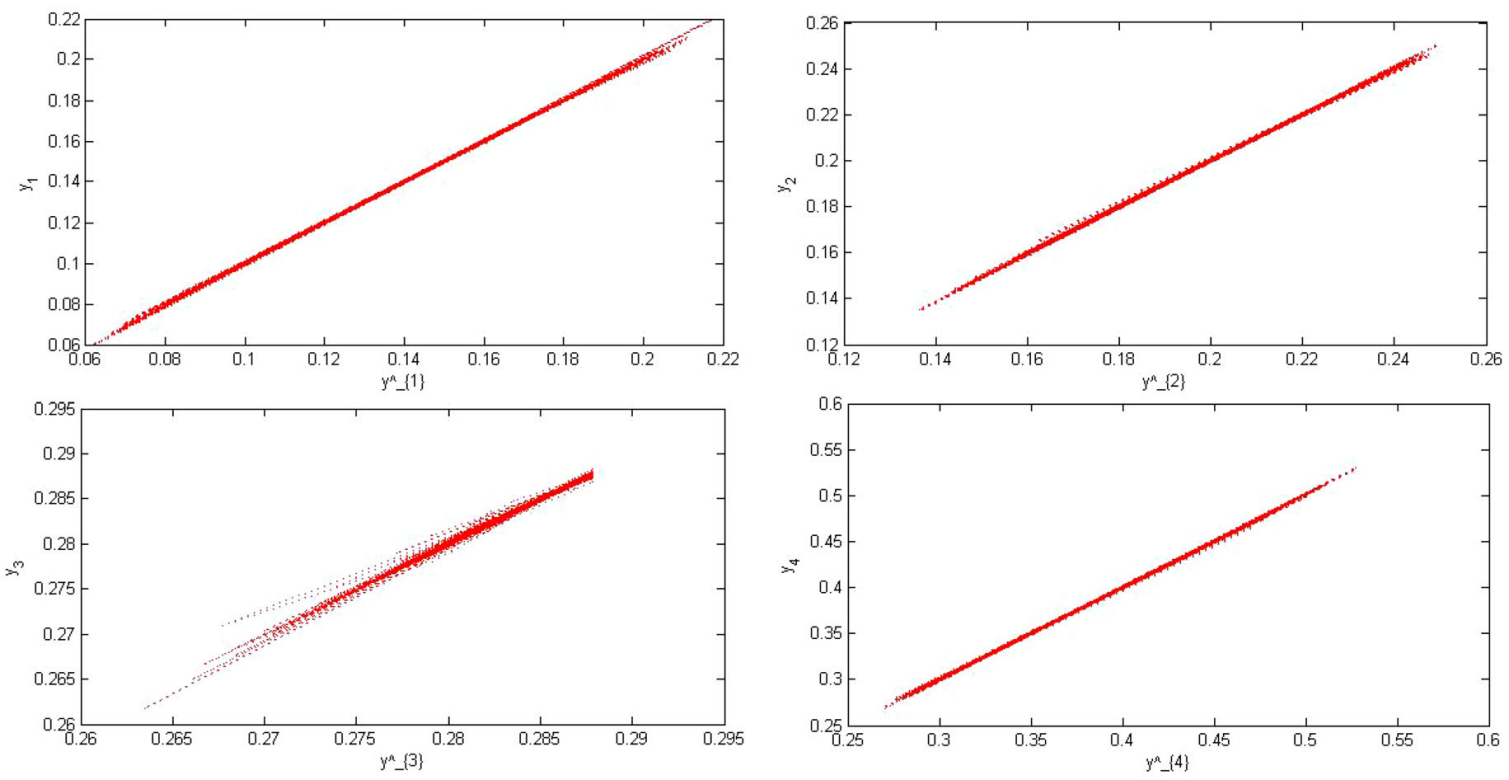
Point cloud of the real output versus the estimated output

The evolutions of the real output $\pi$ and the estimated output *U* (Sect. 2.2) are shown in Fig. 2, and the point cloud of the real output versus the estimated output is given in Fig. 3.

### Sensitivity indices

The sensitivity indices of first order, $S_{1}$ and $S_{2}$, corresponding, respectively, to the arrival rate, $X_{1}= \lambda$, and the service rate, $X_{2}=\mu$, are estimated by (29). Similarly, the index of second order, $S_{12}$, due to the interaction between $\lambda$ and $\mu$, is estimated by (30). Finally, the total indices, $S_{T_{1}}$ and $S_{T_{2}}$, are estimated by (31). These indices are presented in Table 5.

**Table 2:** Values of sensitivity indices for each components of the stationary distribution corresponding at each variable

	Indices	$\pi _{1}$	$\pi _{2}$	$\pi _{3}$	$\pi _{4}$
$\lambda$	$S_{1}$	0.492632321831871	0.761827485935267	0.535225199339738	0.806118443425170
$\mu$	$S_{2}$	0.327092547010556	0.204661797417836	0.430816529889050	0.176927721798758
	$S_{12}$	0.180275131157572	0.033510716646896	0.033958270771211	0.025991651709713
	$S_{T_{1}}$	0.672907452989443	0.795338202582163	0.569183470110949	0.832110095134883
	$S_{T_{2}}$	0.507367678168128	0.238172514064732	0.464774800660261	0.202919373508471

We observe that the parameter $\lambda$ has the highest sensitivity index for each component of the stationary distribution. Then comes the parameter $\mu$. This explains the importance of the contribution of the arrival rate on the performance of the system. Moreover, the total sensitivity indices are close to the first-order indices, since the contribution due to the interaction between $\lambda$ and $\mu$ is low. This means that the contributions of $\lambda$ and $\mu$ are additive.

### Propagation of epistemic uncertainty in the model

In this section, we simplify the model by fixing the least influential parameters ($\mu =\mu _{0}$).

**Fig. 4:**
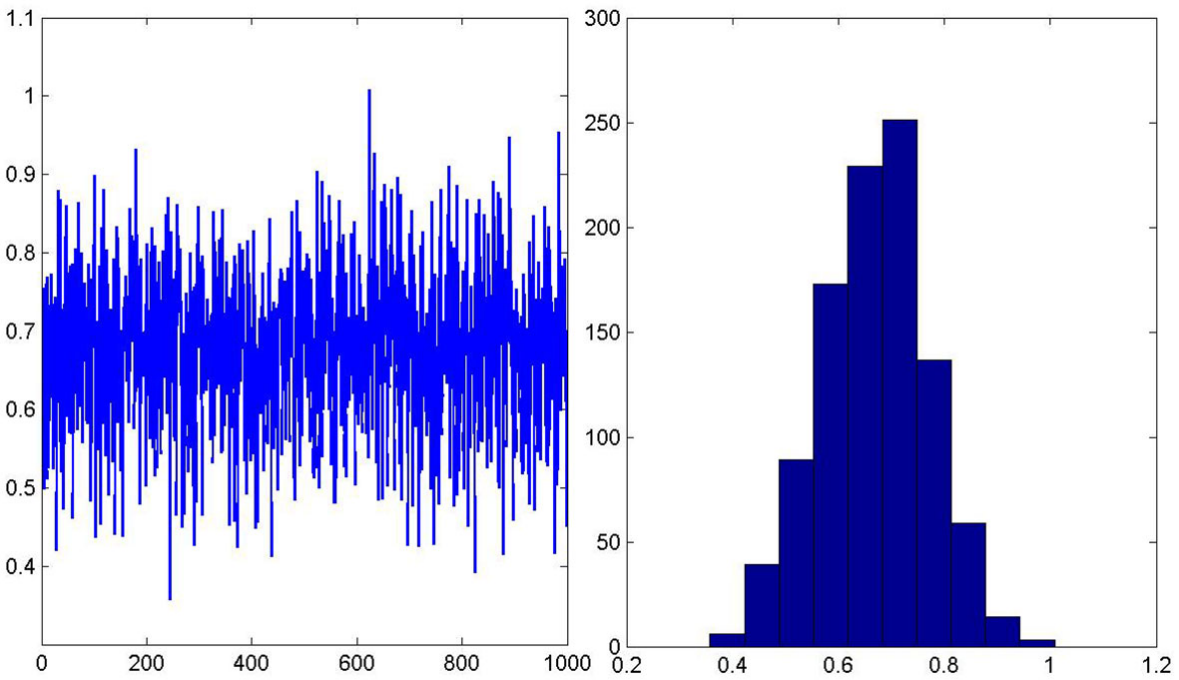
Histogram and plot for the uncertain parameter $\lambda$ with ${\overline{\lambda }}=2/3$ and $\sigma =0.1$ for $\varepsilon \sim N(0,1)$

We will approximate the expectation and the variance of the stationary distribution as a function of the parameter $\lambda$ which is obtained under epistemic uncertainty. $\lambda$ can be written as follows:

35 $$\begin{aligned} \lambda = {\overline{\lambda }} + \sigma \varepsilon , \end{aligned}$$

where ${\overline{\lambda }}$ is the mean of arrival rate $\lambda$, $\sigma$ its standard deviation and $\varepsilon$ is the random noise inflicted on $\lambda$. This noise is called exogenous noise and follows the standard normal distribution. Figure 4 represents histogram and plot for the parameter $\lambda$ given in (35) corresponding to the arrival rate.

The function $\pi _{\ell }, \ell =1,\ldots ,N$ where $\lambda$ is given in (35), is approximated by a polynomial chaos [(see (27)]

36 $$\begin{aligned} \pi _{\ell }(\lambda )=\sum _{j=0}^{P_{d}^{p}}{\widehat{b}}_{j}^{\ell }\psi _{j}(\lambda ), \quad \ell =1,\ldots ,N. \end{aligned}$$

The expectation and the variance of $\pi _{\ell }$ for each $\ell =1,\ldots ,N$ are given by

37 $$\begin{aligned} {\mathbf {E}}(\pi _{\ell }(\lambda ))={\mathbf {E}}\left( \sum _{j=0}^{P_{d }^{p}}{\widehat{b}}_{j}^{\ell }\psi _{j}(\lambda )\right) = \sum _{j=0}^{P_{d}^{p}}{\widehat{b}}_{j}^{\ell }{\mathbf {E}}(\psi _{j}(\lambda )), \quad \ell =1,\ldots ,N \end{aligned}$$

and

38 $$\begin{aligned} {\mathbf {V}}(\pi _{\ell }(\lambda ))= & {} {\mathbf {V}}\left( \sum _{j=0}^{P_{d}^{p}} {\widehat{b}}_{j}^{\ell }\psi _{j}(\lambda )\right) \nonumber \\= & {} \sum _{j=0}^{P_{d}^{p}}({\widehat{b}}_{j}^{\ell })^2{\mathbf {V}} (\psi _{j}(\lambda ))+2\sum _{0\le j \le t\le P_{d}^{p}} ({\widehat{b}}_{j}^{\ell })({\widehat{b}}_{t}^{\ell }) {\mathbf {COV}}(\psi _{j}(\lambda ),\psi _{t}(\lambda ))\nonumber \\= & {} \sum _{j=0}^{P_{d}^{p}}({\widehat{b}}_{j}^{\ell })^2{\mathbf {V}} (\psi _{j}(\lambda ))+2\sum _{j=0}^{P_{d}^{p}}\sum _{t=0}^{P_{d}^{p}}( {\widehat{b}}_{j}^{\ell })({\widehat{b}}_{t}^{\ell }) {\mathbf {COV}}(\psi _{j}(\lambda ),\psi _{t}(\lambda )),\quad \ell =1,\ldots ,N. \end{aligned}$$

Since

39 $$\begin{aligned} {\mathbf {COV}}(\psi _{j}(\lambda ),\psi _{t}(\lambda ))={\mathbf {E}} (\psi _{j}(\lambda ).\psi _{t}(\lambda ))-{\mathbf {E}}(\psi _{j}(\lambda )) {\mathbf {E}}(\psi _{t}(\lambda )), \end{aligned}$$

(38) becomes as

40 $$\begin{aligned} {\mathbf {V}}(\pi _{\ell }(\lambda ))= & {} \sum _{j=0}^{P_{d}^{p}} ({\widehat{b}}_{j}^{\ell })^2{\mathbf {V}}(\psi _{j}(\lambda )) +2\sum _{j=0}^{P_{d}^{p}}\sum _{t=0}^{P_{d}^{p}}({\widehat{b}}_{j}^{\ell }) ({\widehat{b}}_{t}^{\ell }) [{\mathbf {E}}(\psi _{j}(\lambda ).\psi _{t}(\lambda ))-{\mathbf {E}}(\psi _{j}(\lambda )) .{\mathbf {E}}(\psi _{t}(\lambda ))],\nonumber \\&\ell =1,\ldots ,N. \end{aligned}$$

In particular, we take $N=4$, the desired degree for the polynomials is fixed to $p=3$. According to (10), the number of coefficients ${\widehat{b}}_{j}^{\ell }, \ell =1,\ldots ,N$ is equal to 4, and then, the polynomials $\psi _{j}(\lambda )$ are: $\psi _{0}(\lambda )=1$, $\psi _{1}(\lambda )=\lambda$, $\psi _{2}(\lambda )=\lambda ^2-1$, $\psi _{3}(\lambda )=\lambda ^3-3\lambda$.

The formula (36) becomes

41 $$\begin{aligned} \pi _{\ell }(\lambda )={\widehat{b}}_{0}^{\ell }\psi _{0}(\lambda ) +{\widehat{b}}_{1}^{\ell }\psi _{1}(\lambda )+{\widehat{b}}_{2}^{\ell }\psi _{2}(\lambda ) +{\widehat{b}}_{3}^{\ell }\psi _{3}(\lambda ),\quad \ell =1,\ldots ,N; \end{aligned}$$

the expectation and the variance of each component of the stationary distribution are given by

42 $$\begin{aligned} {\mathbf {E}}(\pi _{\ell }(\lambda ))= & {} {\widehat{b}}_{0}^{\ell }{\mathbf {E}} (\psi _{0}(\lambda ))+{\widehat{b}}_{1}^{\ell }{\mathbf {E}}(\psi _{1}(\lambda )) +{\widehat{b}}_{2}^{\ell }{\mathbf {E}}(\psi _{2}(\lambda )) +{\widehat{b}}_{3}^{\ell }{\mathbf {E}}(\psi _{3}(\lambda ))\nonumber \\= & {} {\widehat{b}}_{0}^{\ell }+{\widehat{b}}_{1}^{\ell }{\overline{\lambda }} +{\widehat{b}}_{2}^{\ell } ({\overline{\lambda }}^2+\sigma ^2-1)+{\widehat{b}}_{3}^{\ell } ({\overline{\lambda }}^3+3{\overline{\lambda }}\sigma ^2 -3{\overline{\lambda }}),\quad \ell =1,\ldots ,N \end{aligned}$$

and

43 $$\begin{aligned} {\mathbf {V}}(\pi _{\ell }(\lambda ))= & {} ({\widehat{b}}_{0}^{\ell })^2{\mathbf {V}} (\psi _{0}(\lambda ))+({\widehat{b}}_{1}^{\ell })^2{\mathbf {V}}(\psi _{1}(\lambda ))+ ({\widehat{b}}_{2}^{\ell })^2{\mathbf {V}}(\psi _{2}(\lambda ))+({\widehat{b} }_{3}^{\ell })^2{\mathbf {V}}(\psi _{3}(\lambda ))\nonumber \\&+ 2[{\widehat{b}}_{0}^{\ell }{\widehat{b}}_{1}^{\ell }{\mathbf {COV}}(\psi _{0} (\lambda ),\psi _{1}(\lambda ))+{\widehat{b}}_{0}^{\ell }{\widehat{b} }_{2}^{\ell }{\mathbf {COV}}(\psi _{0}(\lambda ),\psi _{2}(\lambda )) +{\widehat{b}}_{0}^{\ell }b_{3}^{\ell }{\mathbf {COV}}(\psi _{0}(\lambda ), \psi _{3}(\lambda ))\nonumber \\&+{\widehat{b}}_{1}^{\ell } {\widehat{b}}_{2}^{\ell }{\mathbf {COV}}(\psi _{1}(\lambda ),\psi _{2}(\lambda ))+ {\widehat{b}}_{1}^{\ell }{\widehat{b}}_{3}^{\ell }{\mathbf {COV}}(\psi _{1} (\lambda ),\psi _{3}(\lambda ))\nonumber \\&+{\widehat{b}}_{2}^{\ell }b_{3}^{\ell }{\mathbf {COV}}(\psi _{2}(\lambda ), \psi _{3}(\lambda ))],\quad \ell =1,\ldots ,N, \end{aligned}$$

where

44 $$\begin{aligned}&{\mathbf {V}}(\psi _{0}(\lambda ))=0, {\mathbf {V}}(\psi _{1}(\lambda ))=\sigma ^2 \end{aligned}$$

45 $$\begin{aligned}&{\mathbf {V}}(\psi _{2}(\lambda ))=2\sigma ^4+4{\overline{\lambda }}^2\sigma ^2 \end{aligned}$$

46 $$\begin{aligned}&{\mathbf {V}}(\psi _{3}(\lambda ))=9{\overline{\lambda }}^4\sigma ^2+36{\overline{\lambda }}^2\sigma ^4-18{\overline{\lambda }}^2\sigma ^2 +15\sigma ^6-9\sigma ^2-18\sigma ^4 \end{aligned}$$

47 $$\begin{aligned}&{\mathbf {COV}}(\psi _{0}(\lambda ),\psi _{1}(\lambda ))={\mathbf {COV}}(\psi _{0}(\lambda ),\psi _{2}(\lambda ))= {\mathbf {COV}}(\psi _{0}(\lambda ),\psi _{3}(\lambda ))=0 \end{aligned}$$

48 $$\begin{aligned}&{\mathbf {COV}}(\psi _{1}(\lambda ),\psi _{2}(\lambda ))=2{\overline{\lambda }}\sigma ^2 \end{aligned}$$

49 $$\begin{aligned}&{\mathbf {COV}}(\psi _{1}(\lambda ),\psi _{3}(\lambda ))= 3{\overline{\lambda }}^2\sigma ^2+3\sigma ^4-3\sigma ^2 \end{aligned}$$

50 $$\begin{aligned}&{\mathbf {COV}}(\psi _{2}(\lambda ),\psi _{3}(\lambda ))=6{\overline{\lambda }}^3\sigma ^2+12{\overline{\lambda }}\sigma ^4 +3{\overline{\lambda }}\sigma ^2+3{\overline{\lambda }}^3-3{\overline{\lambda }}^2-3\sigma ^2. \end{aligned}$$

The coefficients ${\widehat{b}}_{j}^{\ell },j=0,1,2,3; \ell =1,2,3,4$ are estimated by (18).

For our numerical experiment, we set ${\overline{\lambda }}=2/3$, $\mu _{0}=1$, $\sigma =0.1$, Furthermore, we validate the numerical results by Monte Carlo simulation.

**Table 3:** Expectation of $\pi _{\ell }$, $\ell =1, 2, 3, 4$: polynomial chaos approximation and simulation results for M/M/1 queue with finite capacity ${N} =4$

Expectation	$\pi _{1}$	$\pi _{2}$	$\pi _{3}$	$\pi _{4}$
Chao polynomials	0.189424829068092	0.239463544984460	0.273643697175369	0.297467928716369
Simulation	0.189633158903072	0.239540479209026	0.273569252079986	0.297257109807915

**Table 4:** Variance of $\pi _{\ell }$, $\ell =1, 2, 3, 4$: polynomial chaos approximation and simulation results for M/M/1 queue with finite capacity $N=4$

Variance	$\pi _{1}$	$\pi _{2}$	$\pi _{3}$	$\pi _{4}$
Chao polynomials	0.010061262289655	0.003080593943585	0.002750465866030	0.012768052558072
Simulation	0.000559963015807	0.000614049935349	0.000777697015809	0.001124858491119

#### Performance measures expectation

Let S be the number of customers in the system that is given as a function of the random variable $\lambda$ [see (35)], and it is defined by

51 $$\begin{aligned} S_{\lambda }=S_{{\overline{\lambda }}+\sigma \varepsilon }=\sum _{\ell =1}^{N} \ell ~\pi _{\ell }(\lambda )=\sum _{\ell =1}^{N} \ell ~\pi _{\ell }({\overline{\lambda }}+\sigma \varepsilon ), \end{aligned}$$

where $\pi _{\ell }(\lambda )$ is given in (41). We define the average number of customers and the average waiting time of an arbitrary customers in the system respectively as follows (see [1]):

52 $$\begin{aligned} L={\mathbb {E}}( S_{\lambda })=\sum _{\ell =1}^{N}\ell ~{\mathbb {E}}(\pi _{\ell }(\lambda )), \end{aligned}$$

and

53 $$\begin{aligned} W=\int \frac{1}{\lambda ^{\star }}f_{\lambda }(\lambda )\text {d}\lambda , \end{aligned}$$

where $f_{\lambda }$ is the probability density function of the random variable $\lambda$ and $\lambda ^{\star }$ is the effective arrival rate which is given by

54 $$\begin{aligned} \lambda ^{\star }=\lambda (1-\pi _{N}). \end{aligned}$$

Now, we calculate both performance measures *L* and *W*, and we will compare them with Monte Carlo simulation. The results obtained are given in Table 5.

**Table 5:** Performance measures: polynomial chaos approximation and simulation results for M/M/1 queue with finite capacity ${N}=4$

Performance measures	*L*	*W*
Polynomial chaos	2.679154725428595	5.738811311896458
Simulation	2.678450312792744	5.732499651071908

The comparison of the obtained results shows that the performance of the system calculated by polynomial chaos approximation are very close to that obtained by simulation. This explains that a third-order polynomial chaos yields a good approximation of the stationary distribution.

## Data example on the coronavirus (COVID-19) in Algeria

Many countries are currently dealing with the COVID-19 epidemic and are searching for an exit strategy, such that life in society can return to normal. The load has increased tremendously in the hospitals where the resources are minimal. The queuing model is applied to identify the queue time of the patients in hospitals for the identification and confirmation of disease.

M/M/1/N model is adopted for the study, to model the queue time for the patients in a single-server system. This model is defined as the M/M/1 model with the finite capacity, *N*. In this model, $\lambda$ is defined as the arrival rate of patients (COVID patients only); $\mu$ is the mean service time of patients.

The patient approach to the hospitals for the treatment follows the Poisson probability distribution function at time *t*. The Poisson probability distribution function is defined for the $\lambda$ (patients) per unit *t* (time), and all the patients will be served at a first-come, first-served (FCFS) basis. The time taken to treat the patients would be applied exponentially. The server time is assessed as $\mu$ (patients) per unit *t* (time). The service rate is not depending upon the arrival rate of patients, where the service rate is the time required for the treatment.

Data of COVID-19 patients were taken for the study in the period of 16th June 2021–8th August 2021 from the website (https://www.coronatracker.com).

### Results and discussion

**Fig. 5:**
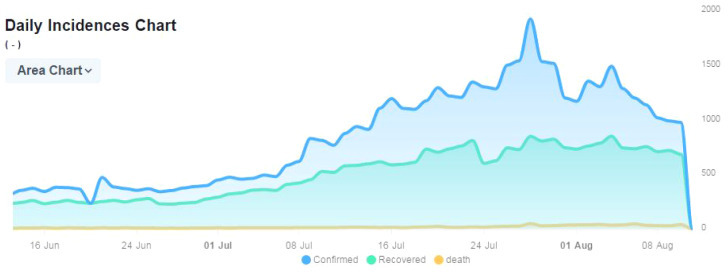
Daily confirmed, recovered, and fatalities of Corona patients in Algeria

The model has been designed specifically for the estimation of processing time of patients diagnosed with the novel Coronavirus. The number of cases of patients suffering from the Corona has increased exponentially, The single-server finite queuing model is designed to compute the time required for each patient. The MATLAB software is used to design the model. The patient data are used for testing the model using the arrival rate of patients ($\lambda$) as 3, and the service rate of patients ($\mu$) per hour as 7. Figure 5 shows the increase in the daily cases of coronavirus in Algeria, and the number of recovered patients and casualties occurred due to COVID-19. It is visible from the graph that the rate of infections is similar to the rate of recovery of patients. However, the number of Corona positive cases is increasing at a rapid rate. Whereas, the fatalities were found low throughout the period.

The single-server queuing model is designed and results obtained based on the stationary distribution of the system are shown in Table 6. The average number of patients in the system is found extensively high as 17.1131786315087, with the average time devoted by the patient in the system which is found as 6.348107106018424 min.

**Table 6:** Performance measurement of single-server queuing model

*N*	${\overline{\lambda }}$	$\mu$	$P_{0}$	$\rho$	*L*	*W*
100	3	7	0.024517395616742	0.428571	17.1131786315087	6.348107106018424

For a large and more general study, one can consider the multi-server queuing model (M/M/s/N) to identify the optimal number of server requirement with the less waiting time and minimum time spent by the patient in the system (see [16]).

## Conclusion

In this paper, a sensitivity analysis approach for an queueing model with parameters that follow a normal distribution has been proposed. The sensitivity indices are estimated by the method based on the PC decomposition of the output of the model. The advantage of this approach is that the number of evaluations of the model is few elevated and the indices are directly obtained from the coefficients of the PC. In addition, the proposed approach was applied to M/GI/1 queuing system, where we have demonstrated that the arrival rate is the most influential parameter on the system and we are fixed the service rate. We were able to estimate the values of each component of the stationary distribution while characterizing its expectation and its variance. Some important performance measures of the queueing model such as the mean system length and the mean waiting time of an arbitrary customer are discussed and evaluated under propagation of epistemic uncertainty in the model input parameter. Moreover, numerical results were presented and compared to the corresponding Monte Carlo simulations ones. The model adopted depends on two parameters; it would be interesting to study more complex models with similar approach.
